# Carbohydrate catabolic flexibility in the mammalian intestinal commensal *Lactobacillus ruminis* revealed by fermentation studies aligned to genome annotations

**DOI:** 10.1186/1475-2859-10-S1-S12

**Published:** 2011-08-30

**Authors:** Michelle M O’ Donnell, Brian M Forde, B Neville, Paul R Ross, Paul W O’ Toole

**Affiliations:** 1 Teagasc Food Research Centre, Moorepark, Fermoy, Co. Cork, Ireland; 2 Department Microbiology, University College Cork, Ireland

## Abstract

**Background:**

*Lactobacillus ruminis* is a poorly characterized member of the *Lactobacillus salivarius* clade that is part of the intestinal microbiota of pigs, humans and other mammals. Its variable abundance in human and animals may be linked to historical changes over time and geographical differences in dietary intake of complex carbohydrates.

**Results:**

In this study, we investigated the ability of nine *L. ruminis* strains of human and bovine origin to utilize fifty carbohydrates including simple sugars, oligosaccharides, and prebiotic polysaccharides. The growth patterns were compared with metabolic pathways predicted by annotation of a high quality draft genome sequence of ATCC 25644 (human isolate) and the complete genome of ATCC 27782 (bovine isolate). All of the strains tested utilized prebiotics including fructooligosaccharides (FOS), soybean-oligosaccharides (SOS) and 1,3:1,4-β-D-gluco-oligosaccharides to varying degrees. Six strains isolated from humans utilized FOS-enriched inulin, as well as FOS. In contrast, three strains isolated from cows grew poorly in FOS-supplemented medium. In general, carbohydrate utilisation patterns were strain-dependent and also varied depending on the degree of polymerisation or complexity of structure. Six putative operons were identified in the genome of the human isolate ATCC 25644 for the transport and utilisation of the prebiotics FOS, galacto-oligosaccharides (GOS), SOS, and 1,3:1,4-β-D-Gluco-oligosaccharides. One of these comprised a novel FOS utilisation operon with predicted capacity to degrade chicory-derived FOS. However, only three of these operons were identified in the ATCC 27782 genome that might account for the utilisation of only SOS and 1,3:1,4-β-D-Gluco-oligosaccharides.

**Conclusions:**

This study has provided definitive genome-based evidence to support the fermentation patterns of nine strains of *Lactobacillus ruminis*, and has linked it to gene distribution patterns in strains from different sources. Furthermore, the study has identified prebiotic carbohydrates with the potential to promote *L. ruminis* growth *in vivo*.

## Background

Immediately following birth, humans are colonised by a variety of bacteria which form the gastrointestinal tract microbiota [1]. Lactic Acid bacteria (LAB), which include *Lactobacillus* spp., are a subdominant element of the microbiota of humans and animals [2].

*Lactobacillus ruminis* is a LAB which is part of the autochthonous microbiota in the intestines of both humans [3], and pigs [4] and it has also been isolated from the bovine rumen [5]. *L. ruminis* is a low G+C Gram positive bacillus [6]. It is a candidate probiotic organism (see below), since it has been reported to have immunomodulatory characteristics [7], specifically the ability to induce Nuclear Factor Kappa B (NF-κB) in the absence of lipopolysaccharide production and to activate Tumour Necrosis Factor alpha (TNFα) production in THP-1 monocytes [7]. Unusually, some strains of *L. ruminis* are motile [5]. Limited studies have identified some of the carbohydrates utilised by *L. ruminis* which include cellobiose and raffinose [5,6,8]. However, little information is available about the fermentation of oligosaccharides/prebiotics by *Lactobacillus ruminis*.

There is growing interest in modulating the human microbiota using dietary supplements including probiotics and prebiotics. Probiotics are defined as “live microorganisms which when administered in adequate amounts confer a health benefit on the host” [9]. However, maintained ingestion of probiotic cultures is generally required to sustain the probiotic effect, with only some of the inoculum surviving gastrointestinal transit, and the vast majority of surviving bacteria shed days after ingestion [10]. For this reason there has been an increasing research effort expended in the area of prebiotics in order to extend the persistence of particular bacteria (mainly bifidobacteria) in the intestine. Prebiotics are “selectively fermented ingredients that result in specific changes in the composition and/or activity of the gastrointestinal microbiota, thus conferring benefit(s) upon host health” [11]. To be considered a prebiotic, the compound has to resist hydrolysis by gastrointestinal tract enzymes and pass into the large intestine, where ideally it promotes the growth of commensal bacteria [12]. The fermentation of prebiotics in the colon is largely influenced by the type of sugar monomer, the degree of polymerisation and the nature of the glycosidic bonds between the sugar moieties [13]. The constituent sugars of the majority of prebiotics are monosaccharides such as glucose, fructose, galactose and xylose [14]. The degree of polymerisation (DP) of prebiotics can vary from as low as two for lactulose and in excess of 23 for chicory-derived inulin [15]. Humans lack the gastrointestinal enzymes necessary to degrade many of the glycosidic bonds between the sugar units of compounds that are prebiotics, which accounts for their resistance to hydrolysis [14]. A number of enzymes produced by colonic commensal bacteria may hydrolyse these bonds. These glycosyl hydrolase (GH) enzymes include β-Glucosidases, α-Glucosidases, β-Fructofuranosidases, β-Galactosidases and α-Galactosidases [16-18].

Studies of other *Lactobacillus* species have identified a variety of genetic systems that encode the ability to utilize carbohydrates of varying complexity. β-fructofuranosidase is responsible for the hydrolysis of FOS, and this activity was identified in *L. plantarum* WCFS1 [19], *L. acidophilus* NCFM [20], and *L. paracasei* 1195 [21]. β-galactosidases involved in lactose degradation were characterised in *L. sakei*[22], *L. bulgaricus*[23], *L. coryniformis*[24] and *L. reuteri*[25]. β-glucosidase activity (which is responsible for the hydrolysis of 1,4-β-D-Glucans like cellobiose) has been identified in *L. plantarum*[26]. α-galactosidases, which hydrolyse α-galactosides like raffinose, stachyose and melibiose, were identified in *L. plantarum* ATCC 8014 [27] and *L. reuteri*[28]. Moreover, several α-glucosidases have been characterised in *L. brevis*[29], *L. acidophilus*[30] and *L. pentosus*[31].

In this study, we describe the fermentation profiles of nine strains of *Lactobacillus ruminis*. The interpretation of the carbohydrate utilisation profiles generated was complemented by the annotation of carbohydrate utilisation genes in the genomes of *L. ruminis* ATCC 25644 and ATCC 27782.

## Results

### Growth of *L. ruminis* in media containing diverse carbon sources

A carbohydrate utilisation profile for each of nine strains of *L. ruminis* on fifty carbohydrates was established as described in Methods. Additional file 1 summarizes the data, with individual strain data in Additional Files 2, 3, 4, 5, 6, 7, 8, 9, 10. In summary, there was significant variation with respect to carbohydrate fermentation profiles at the strain level. Moderate growth was observed for strains L5 and S21 when grown on α-galactosides (melibiose, raffinose, stachyose) and β-glucosides (β-glucotriose B, cellobiose) (Additional file 1). The majority of bovine isolates could poorly utilize fructooligosaccharides, except for ATCC 27781 with Beneo P95 and Raftilose P95. Moderate growth was observed for the majority of isolates with galactooligosaccharides (GOS, GOS-inulin, lactose, lactulose). All strains were able to ferment β-Glucotriose B, cellobiose, galactose, glucose, maltose, mannose, melibiose, raffinose, stachyose and sucrose (Additional file 1). Some strains showed a distinctly higher ability to utilize specific carbohydrates e.g. fructose by strains L5 and S21, (Additional Files 2 and 3); lactose by strains S23, ATCC 25644 and ATCC 27780T (Additional Files 4, 7 and 8); raffinose by ATCC 27781 (Additional File 8); and Raftilose P95 by strain S36 (Additional File 5).

### Growth and fermentation analysis of human and bovine-derived *L. ruminis* type strains

Table 1 shows the final cell numbers and culture-medium pH values reached for the two strains ATCC 25644 (human isolate) and ATCC 27782 (bovine isolate), in the presence of various carbohydrates and prebiotics for 24 h. *L. ruminis* ATCC 25644 reached the highest cell density (8.9 x 10^8^ cfu/ml) when grown on Raftilose Synergy 1 which coincided with the lowest culture medium pH value of 4.86. ATCC 27782 reached the highest cell density values (2.7x10^8^ cfu/ml) when grown on Beta Glucotriose B, and fermentation resulted in a culture medium pH value of 5.19 following 24 hours incubation. This was far higher than cellobiose, the other beta-glucoside tested, although the final pH of both cultures was very similar, and the medium was buffered in the same way as MRS.

**Table 1:** Growth and fermentation analysis of *L. ruminis* strains ATCC 25644 (human isolate) and ATCC 27782 (bovine isolate).

Carbohydrate type	Carbohydrate	ATCC 25644		ATCC 27782	
		
		Cfu/ml	pH*	Cfu/ml	pH*
	Cellobiose	2.40 x 10^8^	5.21	7.00 x 10^6^	5.13
Disaccharide	Lactulose	3.20 x 10^8^	4.99	0	6.53
	Lactose	2.76 x 10^8^	4.76	0	6.57
					
Monosaccharide	Glucose	4.39 x 10^8^	4.86	1.53 x 10^8^	4.85
					
	Beta Glucotriose B	4.05 x 10^8^	5.17	2.66 x 10^8^	5.19
Oligosaccharide	Raftilose Synergy 1	8.90 x 10^8^	5.01	1.35 x 10^7^	6.04
	Raftilose P95	2.91 x 10^8^	5.28	2.51 x 10^6^	5.42
					
Tetrasaccharide	Stachyose	3.94 x 10^8^	5.13	2.37 x 10^8^	5.11
					
Trisaccharide	Raffinose	3.24 x 10^8^	5.2	1.40 x 10^8^	5.2

*. pH value of culture medium after 24 h growth in indicated carbon source. Values tabulated are the average of two replicates carried out on separate days.

### Annotation of carbohydrate pathways in the *L. ruminis* genome

**Figure 1:**
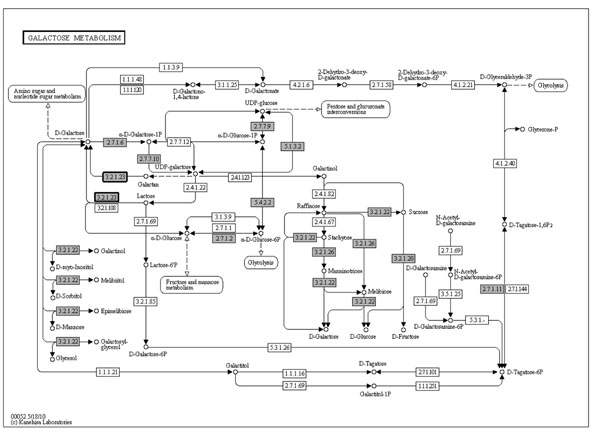
**Galactoside utilisation metabolic map for *L. ruminis* ATCC 25644 and ATCC 27782.** Grey boxes, enzymes present in both ATCC 25644 and ATCC 27782; Grey boxes with emphasised black border, enzymes present in ATCC 25644 and absent from ATCC 27782.

A high-quality draft genome sequence was generated for *L. ruminis* ATCC 25644 and a finished genome sequence was generated for ATCC 27782, as described in Methods. The complete functional and comparative analysis of these genomes will be described elsewhere (Forde *et al.*, in preparation; Neville *et al*., in preparation). A draft sequence of ATCC 25644 has also been generated by the Human Microbiome Project [32]; however it has a different scaffold structure and assembly statistics to that which we generated for ATCC 25644, and for that reason was not used in the current study. The carbohydrate utilisation genes of ATCC 25644 and ATCC 27782 were annotated by manual curation in conjunction with KEGG Automatic Annotation Server (KAAS). *L. ruminis*-specific Kyoto Encyclopaedia of Genes and Genomes (KEGG) maps were generated based upon our annotated genome sequences that we analyzed with KAAS. As a representative example, the galactose metabolic pathway (for both sequenced *L. ruminis* genomes) is presented in Figure 1. It demonstrates the predicted reliance on glycosyl hydrolases to ferment carbohydrates in *L. ruminis* as well as highlighting the fermentable α and β-galactosides.

Sixteen major pathways or systems involved in carbohydrate utilization were annotated in both genomes, and are shown in Additional Files 11, 12, 13, 14, 15, 16, 17, 18, 19, 20, 21, 22, 23, 24, 25, 26. These include those for glycolysis, pentose and glucuronate interconversions, fructose and mannose utilization, starch and sucrose. Of the sixteen pathways identified, eight are considered partial pathways (Additional Files 11, 12, 13, 14, 15, 16, 17, 18, 19, 20, 21, 22, 23, 24, 25, 26).

### Identification of Glycosyl Hydrolases

Glycosyl hydrolases are key to prebiotic utilization, and can also be manipulated to synthesize prebiotics. Twenty glycosyl hydrolases were annotated in the genome of ATCC 25644, and fourteen were annotated in the genome of ATCC 27782. The glycosyl hydrolases include α-amylase (EC 3.2.1.1), endo-1,4-β-xylanase (EC 3.2.1.8), oligo-1,6-glucosidase (EC 3.2.1.10), lysozyme (EC 3.2.1.17), α-glucosidase (EC 3.2.1.20), β-glucosidase (EC 3.2.1.21), α-galactosidase (EC 3.2.1.22), β-galactosidase (EC 3.2.1.23), β-fructofuranosidase (EC 3.2.1.26), β-N-acetylhexosaminidase (EC 3.2.1.52), glucan 1,6-α-glucosidase (EC 3.2.1.70), 6-phospho-β--glucosidase (EC 3.2.1.86) and neopullalanase (EC 3.2.1.135). The majority of these enzymes are present in ATCC 27782 with the exceptions of α-amylase, oligo-1,6-glucosidase and β-galactosidase.

### Identification of putative genes and operons involved in prebiotic utilisation

**Figure 2:**
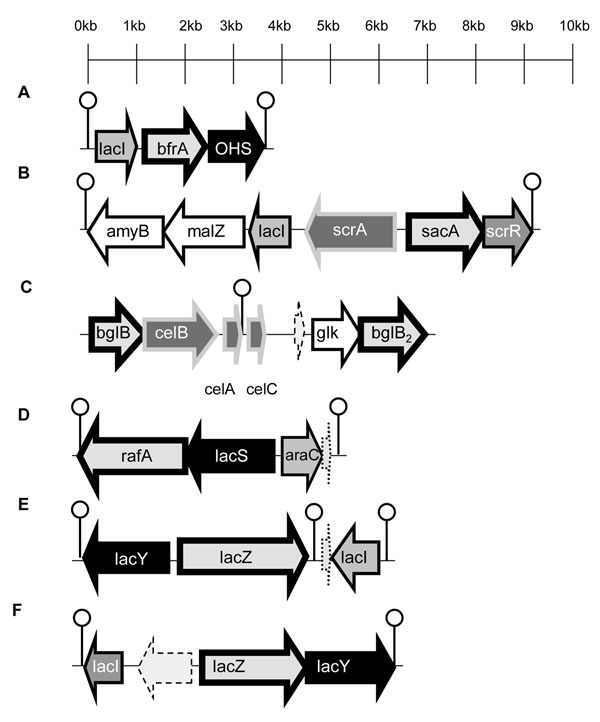
**Putative operons for the predicted utilisation of carbohydrates in *L. ruminis* ATCC 25644.** Predicted substrates are A, FOS; B, Sucrose; C, Cellobiose; D, raffinose; E, lactose/galactose; F, lactose/galactose operon. Light grey arrows with thick black border, glycosyl hydrolase family enzyme; Black arrows, major facilitator superfamily transporters; Medium grey arrows, transcriptional regulators; Dark grey arrows with thick grey border, phosphotransferase system transporters; Lollipops, rho-dependent transcriptional terminators; White arrows with dashed surround, transposases; white arrows with dotted surround, hypothetical proteins; White arrows with black continuous surround, potentially co-transcribed enzymes. Operons B, C and D were also annotated in the ATCC 27782 genome (see Additional File 27: Figure 1).

The sequenced *L. ruminis* genomes were extensively scrutinized to identify putative operons involved in carbohydrate transport and utilisation. Specificity of substrate was based upon manual curation of the annotated region, including reference to BLAST identity to functionally characterized homologues, genetic neighbourhood analysis, and protein motif matching. Six putative prebiotic utilisation operons were annotated in the *L. ruminis* ATCC 25644 genome (human isolate; Figure 2), only three of which were identified in the bovine isolate ATCC 27782 (Additional File 27). Most of the operons are flanked by predicted rho-independent transcriptional terminators (Figure 2), and these operons constitute one to two transcriptional units, with a gene for a LacI-type transcriptional regulator in four of six cases.

We annotated a predicted FOS utilization operon only in the human isolate *L. ruminis* ATCC 25644. β-fructofuranosidase, a Glycosyl hydrolase (GH) family 32 enzyme [16], has been identified as the key enzyme in operons involved in FOS utilisation in other *Lactobacillus* species [19-21]. This activity is predicted to be encoded by the *L. ruminisbfrA* gene, which is linked to a presumptive oligosaccharide symporter gene. The ATCC 25644 genome was also distinguished by having two additional operons for lactose/galactose utilization (Figure 2). The genomes of both strains harboured operons predicted to confer utilization of sucrose, cellobiose and raffinose. As well as the β-fructofuranosidase (*sacA*) in the sucrose operon, genes for an amylopullalanase (*amyB*) and an α-glucosidase (*malZ*) are also contiguous and are potentially co-transcribed with the sucrose operon, but do not have a predicted function in the hydrolysis of sucrose or FOS (Figure 2B).

The cellobiose operon is predicted to be responsible for the transport and hydrolysis of both cellobiose and 1,3:1,4-β-D-Glucan hydrolysates, and in *L. ruminis* it appears to involve two β glucosidases (Figure 2) that belong to the GH1 family of glycosyl hydrolases [16]. The amino acid sequence of BglB and BglB_2_ showed 70% and 77% identity to the β-glucosidases identified in the genomes of *L. helveticus* DPC 4571 and *L. ultunensis* DSM 16047, respectively. The products of the raffinose operon (Fig. 2D; also present in ATCC 27782) are predicted to have the additional ability to breakdown melibiose and stachyose. All of the glycolytic enzymes discussed above lack predicted transmembrane domains (TMD) and therefore most likely require import of their respective substrates.

### Predicted carbohydrate transporters

A relationship exists between the genomic association of genes and the functional interaction of the proteins they encode [33]. To refine our annotation of the carbohydrate utilisation operons, we therefore performed a detailed analysis of the predicted transporter proteins encoded by the contiguous genes. As for hydrolases, specificity of substrate was predicted based upon an integrated analysis of the annotated region, including reference to BLAST identity to functionally characterized homologues, linked genes, and protein motif matching. Putative carbohydrate transporters were analysed with transmembrane prediction software, with 14 and 10 transporters identified in the genome sequences of *L. ruminis* ATCC 25644 and 27782, respectively (Table 2). The predicted carbohydrate transporters belong to the ATP-binding Cassette family (ABC), the Glycoside-Pentoside-Hexuronide cation symporter family (GPH), the Oligosaccharide H^+^ Symporter (OHS) and the Phosphotransferase System (PTS). Transmembrane domain (TMD) numbers are generally indicative of the type of carbohydrate transporter, with some exceptions [34]. ABC transporters have on average 10-12 TMD but this can be highly variable. PTS transporters have been identified with up to 10 TMD (this study). GPH and OHS transporters (both being Major Facilitator Superfamily transporters) generally have 12 TMD [34]. In ATCC 25644, three GPH transporters were identified (Table 2) and these are predicted to transport the β-galactosides (lactose, galactose, lactulose and GOS) and the α-galactosides (raffinose, melibiose and stachyose). However, in ATCC 27782 only one GPH transporter was identified, which was predicted to transport α-galactosides. The OHS identified in the genome of ATCC 25644 is adjacent to a β-fructofuranosidase and may be involved in transporting FOS. Both genomes encode six predicted PTS transporters, which potentially transport mannose, sucrose, fructose, cellobiose and glucose. In both *L. ruminis* genome sequences, four ABC transporters were identified, with the putative substrates identified as mannose and glycerol-3-phosphate. All of the transporters identified in each genome had associated metabolic genes located either upstream or downstream in the genome, and the majority were arranged in operons. Both genomes also encoded proteins for glucose uptake (with TMD counts of 5 and 9 in ATCC 25644 and ATCC 27782, respectively), and a simple sugar transport system permease protein which was predicted to transport monosaccharides like galactose.

**Table 2:** Transmembrane domains (TMD) of the predicted carbohydrate transport proteins in *Lactobacillus ruminis*

Family	Gene	**Locus number**^a^		Predicted substrate	**TMD**^b^	
				
		ATCC 25644	ATCC 27782		ATCC 25644	ATCC 27782
OHS	*lacY*	ANHS_218	-	FOS	12	-
						
GPH	*lacY*	ANHS_744c ANHS_924	-	Lactose, galactose, galactan	12	-
	lacS	ANHS_783	LRC_18250	Raffinose, stachyose, melibiose	12	12
						
	ugpE	ANHS_648	LRC_16940	Glycerol	6	6
ABC	*ugpA*	ANHS_649c	LRC_16950	Glycerol	6	6
	malG	ANHS_839c	LRC_18720	Maltose	6	6
	malF	ANHS_840c	LRC_18730	Maltose	8	8
						
	manY	ANHS_242	LRC_18860	Mannose	7	7
	manZ	ANHS_243	LRC_18850	Mannose	5	4
PTS	*scrA*	ANHS_846c	LRC_18780	Sucrose, FOS	8	8
	fruA	ANHS_1075	LRC_00800	Fructose	9	9
	celB	ANHS_1218	LRC_02240	Cellobiose	10	10
	gluA	ANHS_851c	LRC_18820	Glucose	9	9

a. Locus number in draft genome sequences

b. TMD: predicted trans-membrane domains, as described in Materials & Methods

## Discussion

We consider *L. ruminis* as a candidate probiotic, which we are also investigating as a potential responder for prebiotic/symbiotic supplementation in humans and animals. Several studies have identified *L. ruminis* in the gastrointestinal tract of humans [35-37]. *L. ruminis* was isolated from the bovine rumen [5], from the pig [4,8]], chickens [38], sheep [39], Svalbard reindeer [40], horses [41-43], cats [44,45], dogs [46] and parrots [47]. *L. ruminis* thus appears to be variably present in the microbiota of humans and many domesticated animals.

*L. ruminis* was previously described as a homofermentative bacterium, with the ability to ferment amygdalin, cellobiose, galactose, maltose, mannose, melibiose, raffinose, salicin, sorbitol and sucrose [48]. In the current study, the nine strains of *L. ruminis* were unable to utilise sorbitol as a carbon source. *L. ruminis* has also been reported to have the ability to ferment D-ribose [49]. However, we observed no growth for any of the nine *L. ruminis* strains when cultured in cfMRS supplemented with ribose. ATCC 27782 lacks a transaldolase gene (and the draft genome sequence suggests ATCC 25644 also lacks this gene), which would account for inability to utilise any of the pentose sugars tested. All of the *L. ruminis* strains tested (with the exception of ATCC 27782 which lacks a *lacZ* gene) had strong growth in lactose. This contrasts with a previous study, where moderate growth was recorded on lactose [48]. It has also been reported that *L. ruminis* showed a strain dependent fermentation of starch [50], and very little growth was recorded for any of the strains tested here.

As a species, *L. rumini*s is generally able to ferment prebiotic compounds including FOS, GOS, lactulose, 1,3:1,4 β-D-Glucooligosaccharides, raffinose and stachyose. Only one strain, S36 was capable of (weakly) fermenting the prebiotic disaccharide palatinose. Palatinose is made by enzymatic rearrangement of the glycosidic linkages present in sucrose from an α-1,2-fructoside to an α-1,6-fructoside [51]. This suggests that the catalytic enzymes involved in sucrose utilisation may no longer be able to degrade the α-1,6-fructoside linkage in this disaccharide. The majority of *L. ruminis* strains achieved higher cell densities when grown on the prebiotic carbohydrates raffinose, lactulose, FOS, GOS and stachyose than when grown in other mono- and disaccharide carbohydrates tested. This growth pattern may be attributed to a niche for *L. ruminis* in the lower gastrointestinal tract (GIT). Mono and disaccharides are often unable to resist the hydrolytic action of the upper GIT, unlike prebiotics, and would not therefore be as freely available as carbon sources for *L. ruminis* in the large intestine. Lactulose, a disaccharide derivative of lactose, has previously been shown to support high level growth of other lactobacilli namely *L. rhamnosus*, *L. paracasei* and *L. salivarius*[52]. Lactulose also supported a high level of growth for the majority of *L. ruminis* strains. The β-galactosides lactulose and GOS are predicted to be transported and hydrolysed in ATCC 25644 by LacY and LacZ as part of the lactose operon. Two operons for β-galactoside utilisation were identified in the genome of ATCC 25644; however neither of these operons or any potential genetic determinants could be identified for lactose utilisation in ATCC 27782. The absence of a lactose operon in the genome may suggest an ecological niche adaptation by ATCC 27782 to an environment devoid of milk sugars.

β**-**glucooligosaccharides such as cellobiose are generally transported and hydrolysed using the cellobiose PTS and β-glucosidase enzymes. Both cellobiose and β-glucotriose B are 1,4-β-D-glucooligosaccharides with a similar structure which allows the transport and utilisation of these carbohydrates by the products of the cellobiose operon. The bovine *L. ruminis* isolates, ATCC 27780T, 27781 and 27782 were previously reported to utilise β-glucan hydrolysates as a carbohydrate source [53], and in that study, all bovine isolates utilised β-glucan hydrolysates of DP3, and only ATCC 27780T was unable to utilise DP4 oligosaccharide. ATCC 27781 was distinguished by being able to utilise the highest percentage of both DP3 and DP4 β glucan. We have shown that all the strains tested in this study were able to utilise the DP3 β-glucan hydrolysates to a moderate degree. The bovine isolate ATCC 27780T achieved the highest growth (data not shown) when utilizing β glucan hydrolysate, in contrast to a previous study which identified ATCC 27781 as having the highest percentage utilisation of β-glucan oligosaccharide [53].

	In previous analysis of sixteen *Lactobacillus* species, only *L. acidophilus* L3, *L. acidophilus* 74-2 and *L. casei* CRL431 were able to utilise Raftilose P95, an oligofructose [54]. In the current study, eight strains of *L. ruminis* were capable of utilizing Raftilose P95. In addition, *L. ruminis* was capable of moderate to strong fermentation of Raftilose Synergy 1, an oligofructose-enriched inulin. *L. paracasei* subsp. *paracasei* 8700:2 was previously shown to be the only strain, out of ten strains tested, that was capable of strong growth on Raftilose Synergy 1, while three other species were capable of moderate growth [55]. Based on these comparisons, *L. ruminis* may have a growth advantage over other lactobacilli in the presence of fructooligosaccharides.

A novel β-fructofuranosidase was identified in the genome of *L. ruminis* ATCC 25644 that potentially hydrolyses the linkages present in chicory derived fructooligosaccharides. The cognate transporter OHS was identified only in the strains isolated from humans. Transport of FOS may be transported using the sucrose PTS transporter in the bovine strains ATCC 27780 and 27781. The human isolates of *L. ruminis* apparently use an OHS to transport FOS into the cell. Both sequenced strains likely use the ABC transport system to transport simple carbohydrates like maltose and glycerol. The most populated class of transporter identified was the phosphotransferase system transporter, with six such systems present. However, in *L. ruminis* many of the fermentable carbohydrates including α-galactosides and β-galactosides are predicted to be transported by GPH symporters. GPH transporters contain a C-terminal hydrophilic domain which interacts with the PTS system [34], which may thus be an important regulatory mechanism in *L. ruminis*.

## Conclusions

*Lactobacillus ruminis* is a saccharolytic member of the intestinal microbiota capable of degrading a variety of prebiotics. Genes and operons were identified in the genomes of two sequenced strains for the hydrolysis and transport of the utilisable prebiotics. This work is the first step in the characterisation of carbohydrate metabolism, transportation and regulation in *L. ruminis*. Further studies will focus on the functional characterisation of the putative operons identified in this study and also *in vivo* studies with dietary supplementation by selected carbohydrates. Characterisation of the novel FOS degrading enzyme BfrA may facilitate applications including reverse engineering of the FOS degradation pathway to allow the biosynthesis of a potentially novel fructooligosaccharide.

## Methods

### Bacterial strains and culture conditions

Nine *Lactobacillus ruminis* strains were used in this study, and were obtained courtesy of Prof. Gerald Tannock, University of Otago, New Zealand. Four of these are American Type Culture Collection strains: ATCC 25644 (human isolate), ATCC 27780T, ATCC 27781 and ATCC 27782 (bovine isolates). Five human-derived *L. ruminis* strains, L5, S21, S23, S36 and S38 were also studied. All strains were stored at -80°C in de Man-Rogosa-Sharpe (MRS) broth (Difco, BD, Ireland), supplemented with 25% (vol/vol) glycerol as a cryoprotectant. *Lactobacillus* strains were grown anaerobically on MRS agar plates at 37°C for two days. Growth tests were initiated by growing *Lactobacillus* strains anaerobically in MRS-glucose broth at 37°C overnight and unless otherwise stated, all further incubations were also performed under anaerobic conditions at 37°C.

### Growth medium

Modifications were made to the de Man-Rogosa-Sharpe (MRS) [56] medium by omitting the carbohydrate source (glucose) and meat extract. Carbohydrate-free MRS (cfMRS) was used as a basal growth medium to study the ability of *Lactobacillus ruminis* strains to utilise various carbohydrates, because it contains no additional carbohydrates and lacks Lab Lembco as a source of carbohydrates. The cfMRS medium contained the following components (gL^-1^): bacteriological peptone (Oxoid) 10.0, yeast extract (Fluka) 5.0, sodium acetate (Sigma) 5.0, ammonium citrate (Sigma) 2.0, potassium phosphate (Sigma) 2.0, magnesium sulphate (BDH Chemical) 0.2, Manganese sulphate (BDH Chemical) 0.05. The medium also includes Tween 80 (Sigma) 1 ml litre^-1^. The pH was adjusted to between 6.2 and 6.5 and the medium was sterilised at 121°C for 15 minutes. Carbohydrate-free MRS was unable to support bacterial growth above an OD_600nm_ of 0.1 for any of the strains tested.

### Carbohydrates and prebiotics

Fifty- two carbohydrates were used in this study (Additional file 28). Stock solutions of the 50 carbohydrates were filter-sterilized (0.45μm) (Sarstedt) into the cfMRS basal medium to yield a concentration of 0.5% (v/v) for use in the fermentation tests.

### Growth measurements

The fermentation profiles of the various strains were determined using optical density (OD) measurements. The sterile carbohydrate supplemented MRS media was added to the wells of 96 well microtiter plates. The medium in the wells was inoculated with 1% (v/v) of the overnight bacterial culture in MRS-glucose. The OD values of the 96 well microtiter plate wells were read using a Synergy 2 plate reader (BioTek Instruments, Inc., Vermont, US). The inoculated microtiter plates were incubated anaerobically at 37°C and OD readings were taken before and after a 48 hour period [57]. The mean OD readings, standard deviations and standard errors were calculated using technical triplicate data from biological duplicate experiments.

### *Lactobacillus ruminis* genome sequencing and assembly

The genome sequencing, assembly and detailed annotation of the *L. ruminis* ATCC 27782 and 25644 genomes will be described elsewhere in this volume (Forde *et al*, manuscript in preparation). In brief, a hybrid next-generation strategy generated 28-fold coverage of the ATCC 27782 genome by 454 pyrosequencing, complimented by 217-fold coverage with Illumina paired-end sequences. The assembly of *L. ruminis* ATCC 27782 is a finished genome; the genome assembly of *L. ruminis* ATCC 25644 a high-quality draft [58].

### Bioinformatic analysis and gene annotation

The Artemis program [59] was used to visualise and identify carbohydrate metabolism genes in the genome of *Lactobacillus ruminis* ATCC 25644 and ATCC 27782 [60]. Open reading frames were predicted using Glimmer 3 [61]. Each carbohydrate utilisation enzyme, predicted from opening reading frames (ORF), was assigned a KEGG orthology (KO) identifier by KAAS and graphical representations for each metabolic pathway were generated [62]. The TMHMM 2.0 server was used to predict the transmembrane helices of proteins, which were identified from annotation as putative carbohydrate transporters. THHMM 2.0 uses Hidden Markov models to predict the proteins topology with a high degree of accuracy [63]. TransTermHP [64] was used to predict rho-independent transcriptional terminators. Comparisons to other Lactobacillus genomes were made using data available from both NCBI [65] and KEGG Organisms [66].

## Sequence data availability and accession numbers

The finished genome of ATCC 27782 is available under accession number XXYYZZ123. The draft genome of ATCC 25644 is available under accession number CCGGHHIIUU.

## Competing interests

The authors declare they have no competing interest.

## Authors' contributions

MMOD designed the experiments and drafted the manuscript. BMF carried out the genome sequencing and assembly of both *L. ruminis* genomes and provided the output of both the TMHMM 2.0 server and TransTerm HP. BAN initiated the genome sequencing and participated in assembly of the genomes. PWOT and RPR conceived the study, designed the research, and contributed to writing the manuscript.

## Supplementary Material

Additional file 1 Fermentation profiles for nine *Lactobacillus ruminis* strains Click here for file

Additional file 2 Growth profile for *L. ruminis* strain L5 Click here for file

Additional file 3 Growth profile for *L. ruminis* strain S21 Click here for file

Additional file 4 Growth profile for *L. ruminis* strain S23 Click here for file

Additional file 5 Growth profile for *L. ruminis* strain S36 Click here for file

Additional file 6 Growth profile for *L. ruminis* strain S38 Click here for file

Additional file 7 Growth profile for *L. ruminis* strain ATCC 25644 Click here for file

Additional file 8 **Growth profile for *L. ruminis* strain ATCC 27780**T Click here for file

Additional file 9 Growth profile for *L. ruminis* strain ATCC 27781 Click here for file

Additional file 10 Growth profile for *L. ruminis* strain ATCC 27782 Click here for file

Additional file 11 Glycolysis map representing enzymes present in *L. ruminis* ATCC 25644 or ATCC 27782. Click here for file

Additional file 12 Citrate cycle map representing enzymes present in *L. ruminis* ATCC 25644 or ATCC 27782. Click here for file

Additional file 13 Pentose phosphate pathway map representing enzymes present in *L. ruminis* ATCC 25644 or ATCC 27782. Click here for file

Additional file 14 Pentose and glucuronate interconversions map representing enzymes present in *L. ruminis* ATCC 25644 or ATCC 27782. Click here for file

Additional file 15 Fructose and Mannose metabolism map representing enzymes present in *L. ruminis* ATCC 25644 or ATCC 27782. Click here for file

Additional file 16 Galactose metabolism map representing enzymes present in *L. ruminis* ATCC 25644 or ATCC 27782. Click here for file

Additional file 17 Ascorbate and aldarate metabolism map representing enzymes present in *L. ruminis* ATCC 25644 or ATCC 27782. Click here for file

Additional file 18 Starch and sucrose metabolism map representing enzymes present in *L. ruminis* ATCC 25644 or ATCC 27782. Click here for file

Additional file 19 Amino and nucleotide sugar metabolism map representing enzymes present in *L. ruminis* ATCC 25644 or ATCC 27782. Click here for file

Additional file 20 Inositol Phosphate metabolism map representing enzymes present in *L. ruminis* ATCC 25644 or ATCC 27782. Click here for file

Additional file 21 Pyruvate metabolism map representing enzymes present in *L. ruminis* ATCC 25644 or ATCC 27782. Click here for file

Additional file 22 Glyoxylate and Dicarboxylate metabolism map representing enzymes present in *L. ruminis* ATCC 25644 or ATCC 27782. Click here for file

Additional file 23 Propanoate metabolic map representing enzymes present in *L. ruminis* ATCC 25644 or ATCC 27782. Click here for file

Additional file 24 Butanoate metabolic map representing enzymes present in *L. ruminis* ATCC 25644 or ATCC 27782. Click here for file

Additional file 25 ABC transporters map representing enzymes present in *L. ruminis* ATCC 25644 or ATCC 27782. Click here for file

Additional file 26 Phosphotransferase system map representing enzymes present in *L. ruminis* ATCC 25644 or ATCC 27782. Click here for file

Additional file 27 Operons in the genome of *L. ruminis* ATCC 27782 associated with prebiotic utilisation Click here for file

Additional file 28 Carbohydrates used in this study Click here for file
