# Supplementary material for: Carbohydrate catabolic flexibility in the mammalian intestinal commensal Lactobacillus ruminis revealed by fermentation studies aligned to genome annotations
Source: Microb Cell Fact. 2011 Aug 30;10(Suppl 1):S12. doi: 10.1186/1475-2859-10-S1-S12 (PMC3231919; doi:10.1186/1475-2859-10-S1-S12)
Supplement: Additional file 1 — Fermentation profiles for nine Lactobacillus ruminis strains [file 1475-2859-10-S1-S12-S1.pdf]

Additional Table 1 - Fermentation profiles for nine *Lactobacillus ruminis* strains

		Lactobacillus ruminis strains								
		Human strains						Bovine strains		
**Carbohydrate type**	**Carbohydrate**	**L5**	**S21**	**S23**	**S36**	**S38**	**ATCC 25644**	**ATCC 27780T**	**ATCC 27781**	**ATCC 27782**
Monosaccharides	D-Arabinose	-	-	-	-	-	-	-	-	-
	L-Arabinose	-	-	-	+	+	-	-	-	-
	Fructose	+++	+++	+++	+++	++	+++	++	++	-
	Galactose	++	++	+++	+++	++	++	++	++	++
	Glucose	++	++	+++	+++	++	+++	++	++	++
	Lyxose	-	-	-	-	-	-	-	-	-
	Mannose	++	++	+++	+++	+++	++	+++	+++	++
	Melibiose	+	+	++	++	+	++	++	++	++
	Ribose	-	-	-	-	-	-	-	-	-
	Xylose	-	-	-	-	-	-	-	-	-
Disaccharides	Cellobiose	++	+	++	++	++	++	++	++	++
				+++						
	Lactose	++	++	+	+++	+++	++++	+++	+++	-
	Lactulose	++	++	+++	++	++	+++	+++	++	-
	Maltose	++	++	++	++	++	++	++	++	++
	Sucrose	++	++	+++	+++	++	+++	+++	++	++
	Trehalose	-	-	-	-	-	-	-	-	ND
Trisaccharides	Raffinose	++	++	+++	+++	+++	+++	+++	+++	++
	Melezitose	-	-	-	-	-	-	-	-	ND
Tetrasaccharide	Stachyose	+	++	++	+++	++	++	++	++	++
Oligosaccharides	Beneo P95	++	++	+++	+++	++	+++	+	++	-
	B-Glucotriose (B)[a]	++	++	++	++	++	++	++	++	++
				+++						
	Raftilose P95	++	++	+	+++	+++	+++	+	++	-
	Raftilose Synergy 1	++	++	+++	++	++	+++	-	+	-
	GOS	++	++	+++	++	++	++	++	++	ND
	GOS Inulin	+	+	++	++	++	++	++	++	-
	Palatinose	-	-	-	+	-	-	-	-	ND
	Polydextrose	+	+	-	-	-	-	-	-	-
Polyols	Mannitol	-	-	-	-	-	-	-	-	ND
	Sorbitol	-	-	-	-	-	-	-	-	ND
	Xylitol	-	-	-	-	-	-	-	-	-
Polysaccharides	Sialic acid	-	-	-	-	-	-	-	-	ND
	Siallylactose	-	-	-	-	-	-	-	-	-
	Soluble Starch	-	-	-	-	-	-	-	-	ND
	Xylan Beechwood	-	-	-	+	-	-	-	-	ND
	Xylan Oatspelts	-	-	-	-	-	-	-	-	ND
	Cellulose	-	-	-	-	-	-	-	-	-
	B- Glucan	-	-	-	-	-	-	-	-	ND
	Dextran	-	-	-	-	-	-	-	-	ND
	Dextrin	-	-	-	-	-	-	-	-	ND
	Esculin	-	-	-	-	-	-	-	-	-
	Beneo HP	-	-	-	-	-	-	-	-	ND
	Lichenan	-	-	-	-	-	-	-	-	ND
	Maltodextrin	-	-	-	-	-	-	+	-	ND
	Mannan	-	-	-	-	-	-	-	-	ND
	Methylcellulose	-	-	-	-	-	-	-	-	-
	Raftiline HP	-	-	-	-	-	-	-	-	-
	Raftiline HPX	-	-	-	-	-	-	-	-	ND
	Raftiline ST	++	-	++	++	++	++	-	-	-
Algal sources	Red Powder	+	+	+	+	+	+	+	+	-
	Green Powder	-	-	-	-	-	-	-	-	ND

(-) no growth (OD ≤ 0.1); (+) weak growth (OD 0.1 – 023); (++) moderate growth (OD 0.2 – 0.5); (+++) strong growth (OD 0.5 – 0.8); (++++) very strong growth (OD 0.8 – 1.0); (ND) not determined. a: β-glucan hydrolysate
