# Supplementary material for: Carbohydrate catabolic flexibility in the mammalian intestinal commensal Lactobacillus ruminis revealed by fermentation studies aligned to genome annotations
Source: Microb Cell Fact. 2011 Aug 30;10(Suppl 1):S12. doi: 10.1186/1475-2859-10-S1-S12 (PMC3231919; doi:10.1186/1475-2859-10-S1-S12)
Supplement: Additional file 7 — Growth profile for L. ruminis strain ATCC 25644 [file 1475-2859-10-S1-S12-S7.pdf]

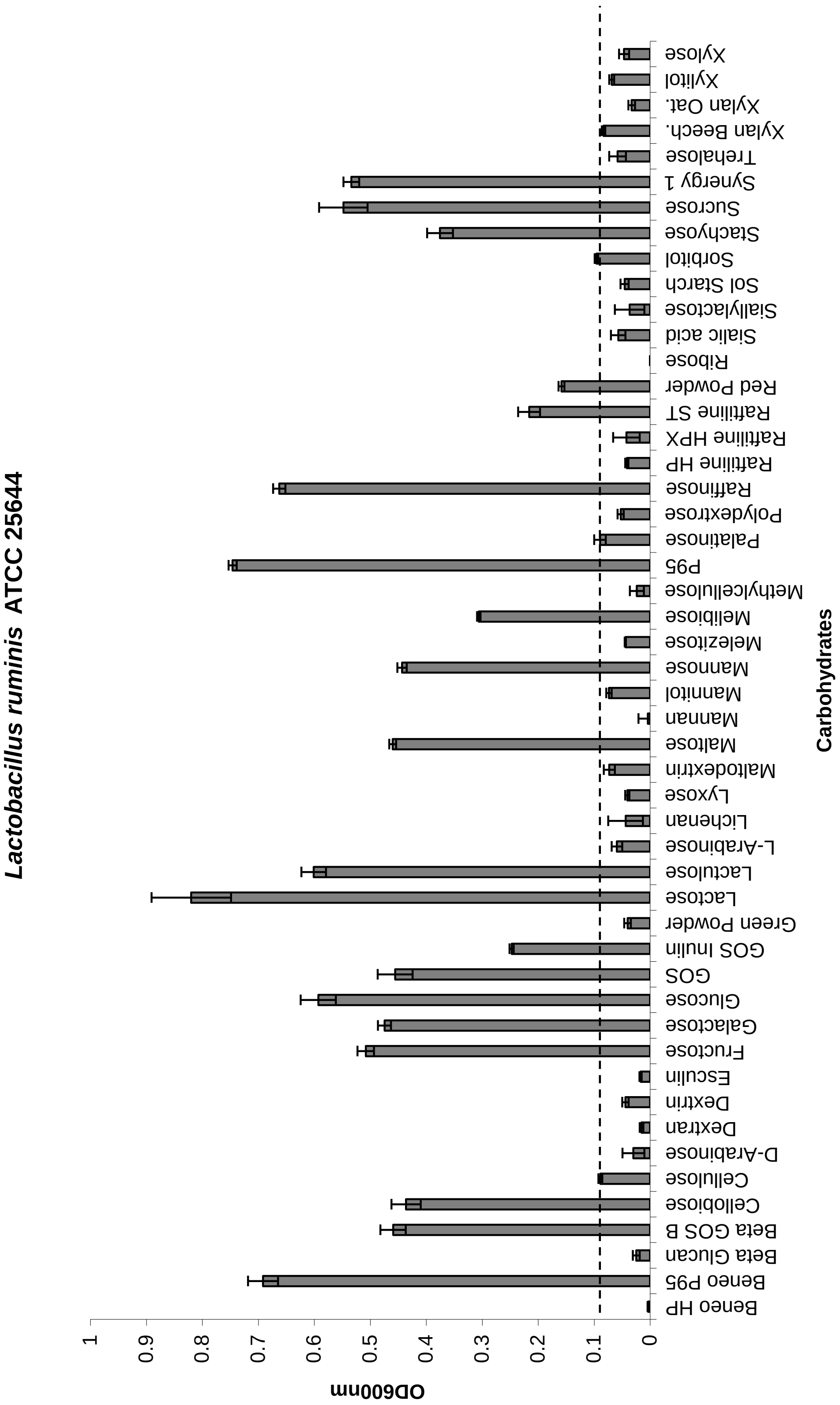

Figure1: Growth profile for *L. ruminis* ATCC 25644. Values plotted are the differences in optical density between time 0 and time 48h ± standard error. Dashed line, cut-off point
