# Supplementary material for: Carbohydrate catabolic flexibility in the mammalian intestinal commensal Lactobacillus ruminis revealed by fermentation studies aligned to genome annotations
Source: Microb Cell Fact. 2011 Aug 30;10(Suppl 1):S12. doi: 10.1186/1475-2859-10-S1-S12 (PMC3231919; doi:10.1186/1475-2859-10-S1-S12)
Supplement: Additional file 27 — Operons in the genome of L. ruminis ATCC 27782 associated with prebiotic utilisation [file 1475-2859-10-S1-S12-S27.pdf]

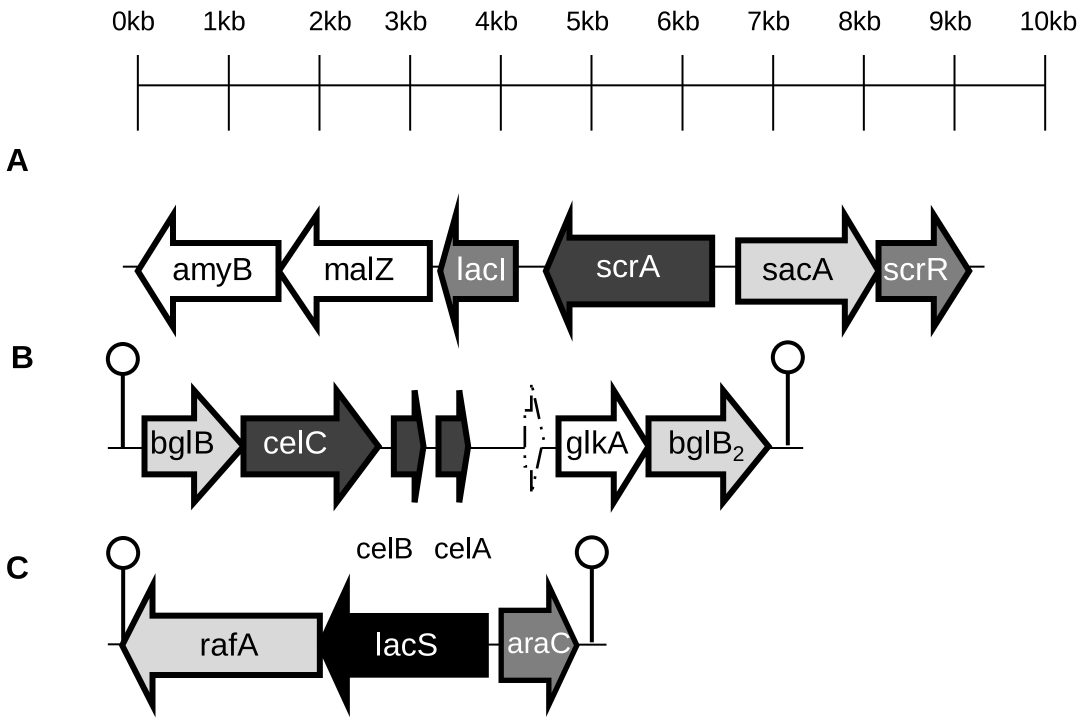

Figure 1: Putative operons predicted to be involved in the utilisation of carbohydrates in ATCC 27782. A, Sucrose operon; B, Cellobiose operon; C, Raffinose operon. Light grey arrows, glycosyl hydrolase family enzyme; Black arrows, major facilitator superfamily transporters; Medium grey arrows transcriptional regulators; Dark grey arrows, phosphotransferase system transporters; Lollipops, rho-independent transcriptional regulators; White arrows with dashed surround, transposases; White arrows with black continuous surround, potentially co-transcribed enzymes.
