# Supplementary material for: Carbohydrate catabolic flexibility in the mammalian intestinal commensal Lactobacillus ruminis revealed by fermentation studies aligned to genome annotations
Source: Microb Cell Fact. 2011 Aug 30;10(Suppl 1):S12. doi: 10.1186/1475-2859-10-S1-S12 (PMC3231919; doi:10.1186/1475-2859-10-S1-S12)
Supplement: Additional file 28 — Carbohydrates used in this study [file 1475-2859-10-S1-S12-S28.pdf]

Additional Table 2 - Carbohydrates used in this study

Carbohydrate type	Name	Source	Degree of Polymerisation
	Glucose	Fisher Scientific	n/a
	Fructose	Sigma Aldrich, Poole, UK	n/a
	Galactose	Sigma Aldrich, Poole, UK	n/a
	D – Arabinose	Sigma Aldrich, Poole, UK	n/a
Monosaccharide	L - Arabinose	Sigma Aldrich, Poole, UK	n/a
	Mannose	Sigma Aldrich, Poole, UK	n/a
	Ribose	Sigma Aldrich, Poole, UK	n/a
	Lyxose	Sigma Aldrich, Poole, UK	n/a
	Xylose	Sigma Aldrich, Poole, UK	n/a
	Sialic acid	Friesland Foods, Zwolle, Netherlands	n/a
	Cellobiose	Sigma Aldrich, Poole, UK	2
	Trehalose	Sigma Aldrich, Poole, UK	2
	Sucrose	Sigma Aldrich, Poole, UK	2
Disaccharide	Maltose	Sigma Aldrich, Poole, UK	2
	Lactose	Sigma Aldrich, Poole, UK	2
	Lactulose	Sigma Aldrich, Poole, UK	2
	Melibiose	Sigma Aldrich, Poole, UK	2
	Palatinose	Sigma Aldrich, Poole, UK	2
Trisaccharide	Melezitose	Sigma Aldrich, Poole, UK	3
	Raffinose	Sigma Aldrich, Poole, UK	3
Tetrasaccharide	Stachyose	Sigma Aldrich, Poole, UK	4
	Soluble Starch	BDH Analar	-
	Maltodextrin	Cargill-Cerestar	Avg. 7
	Polydextrose	Danisco	Avg. 12
	Galactooligosaccharide (GOS)	Friesland Foods ,Zwolle, Netherlands	2 to 8
	GOS inulin	Friesland Foods, Zwolle, Netherlands	Unknown
Oligosaccharide	β-Glucotriose B (β-glucan hydrolysate)	Megazyme, Co. Wicklow, Ireland	3
	Raftilose P95	Orafti, Tienen, Belgium	2 to 8
	Raftilose Synergy 1 (oligofructose enriched inulin)	Orafti, Tienen, Belgium	2 to 8
	Beneo P95	Orafti, Tienen, Belgium	2 to 8
	Dextran	Sigma Aldrich, Poole, UK	-<br>-
	Dextrin	Sigma Aldrich, Poole, UK	-
	β Glucan	Megazyme, Co. Wicklow, Ireland	>100
	Mannan	Megazyme, Co. Wicklow, Ireland	15
	Lichenan	Megazyme, Co. Wicklow, Ireland	80-400
	Beneo HP	Orafti, Tienen, Belgium	>23
Polysaccharide	Raftiline ST	Orafti, Tienen, Belgium	≥10
	Raftiline HPX	Orafti, Tienen, Belgium	≥23
	Raftiline HP	Orafti, Tienen, Belgium	>23
	Xylan from Beechwood	Sigma Aldrich, Poole, UK	100-200
	Xylan from Oatspelts	Sigma Aldrich, Poole, UK	100-200
	Cellulose	Sigma Aldrich, Poole, UK	300-1700
	Methylcellulose	Sigma Aldrich, Poole, UK	-
Polyol	Mannitol	Sigma Aldrich, Poole, UK	n/a
	Sorbitol	Sigma Aldrich, Poole, UK	n/a
	Xylitol	Sigma Aldrich, Poole, UK	n/a
Algal source	Green Powder	Algae derived powder	Unknown
	Red Powder	Algae derived powder	Unknown
Unknown	Esculin	Sigma Aldrich, Poole, UK	-
	Sialyllactose	Friesland Foods, Zwolle, Netherlands	-

(?), Unknown carbohydrate type; (-), unknown degree of polymerisation; n/a, monosaccharides
