# Supplementary material for: Carbohydrate catabolic flexibility in the mammalian intestinal commensal Lactobacillus ruminis revealed by fermentation studies aligned to genome annotations
Source: Microb Cell Fact. 2011 Aug 30;10(Suppl 1):S12. doi: 10.1186/1475-2859-10-S1-S12 (PMC3231919; doi:10.1186/1475-2859-10-S1-S12)
Supplement: Additional file 18 — Starch and sucrose metabolism map representing enzymes present in L. ruminis ATCC 25644 or ATCC 27782. [file 1475-2859-10-S1-S12-S18.pdf]

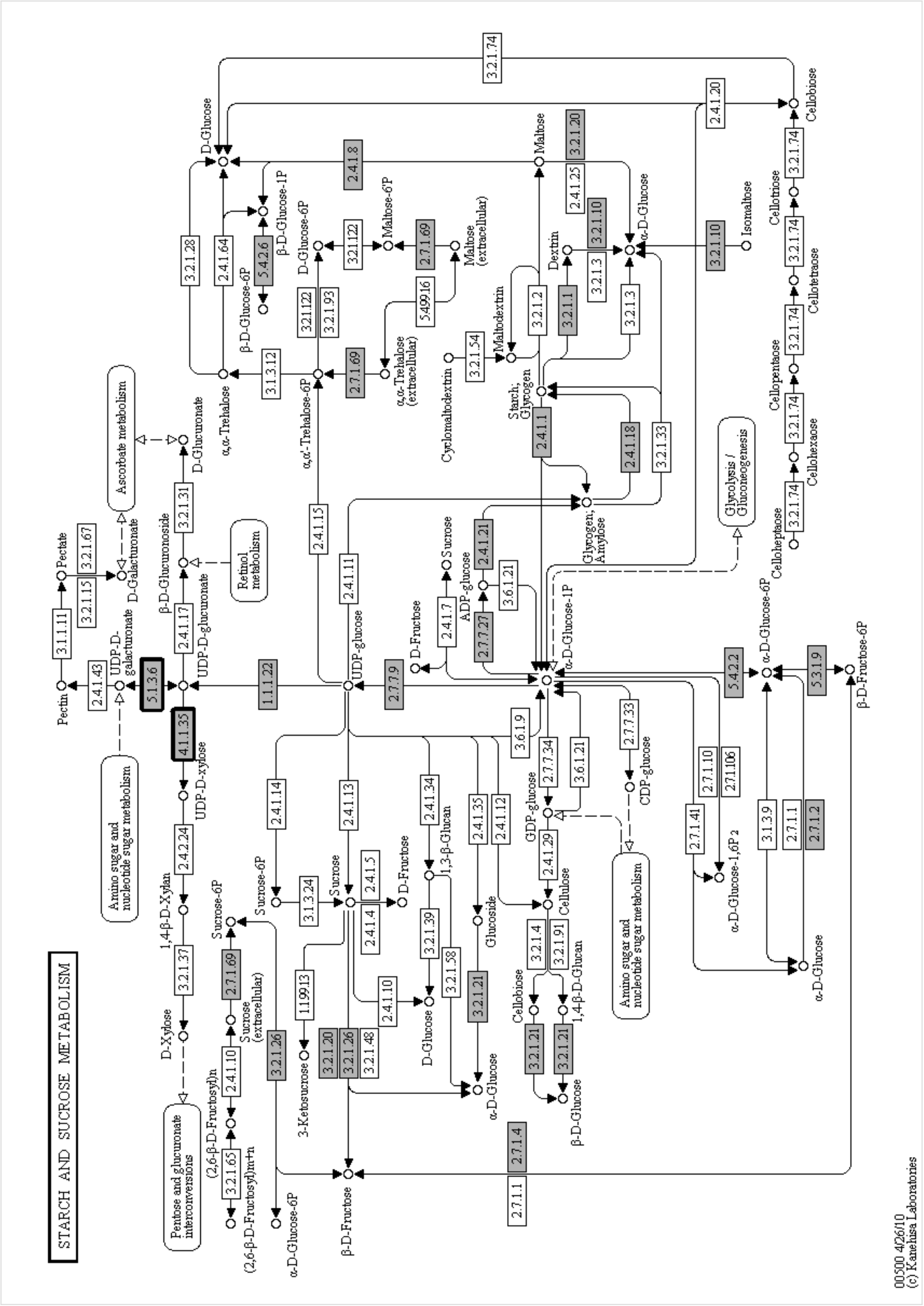

Figure 1: Starch and sucrose metabolism map representing enzymes present in both *L. ruminis* ATCC 25644 and ATCC 27782. Grey boxes, enzymes present in both sequenced strains
